# Biometric Measurement of Anterior Segment: A Review

**DOI:** 10.3390/s20154285

**Published:** 2020-07-31

**Authors:** Bin Liu, Chengwei Kang, Fengzhou Fang

**Affiliations:** 1 Tianjin Key Laboratory for Control Theory & Applications in Complicated Systems, Tianjin University of Technology, Tianjin 300384, China; lbin83@126.com; 2 Centre of Micro/Nano manufacturing Technology (MNMT-Dublin), University College Dublin, D04 V1W8 Dublin 4, Dublin, Ireland; chengwei.kang@ucd.ie; 3 State Key Laboratory of Precision Measuring Technology and Instruments, Centre of Micro/Nano Manufacturing Technology (MNMT), Tianjin University, Tianjin 300372, China

**Keywords:** anterior segment, geometric measurement, corneal topography, tomography

## Abstract

Biometric measurement of the anterior segment is of great importance for the ophthalmology, human eye modeling, contact lens fitting, intraocular lens design, etc. This paper serves as a comprehensive review on the historical development and basic principles of the technologies for measuring the geometric profiles of the anterior segment. Both the advantages and drawbacks of the current technologies are illustrated. For in vivo measurement of the anterior segment, there are two main challenges that need to be addressed to achieve high speed, fine resolution, and large range imaging. One is the motion artefacts caused by the inevitable and random human eye movement. The other is the serious multiple scattering effects in intraocular turbid media. The future research perspectives are also outlined in this paper.

## 1. Introduction

**Figure 1:**
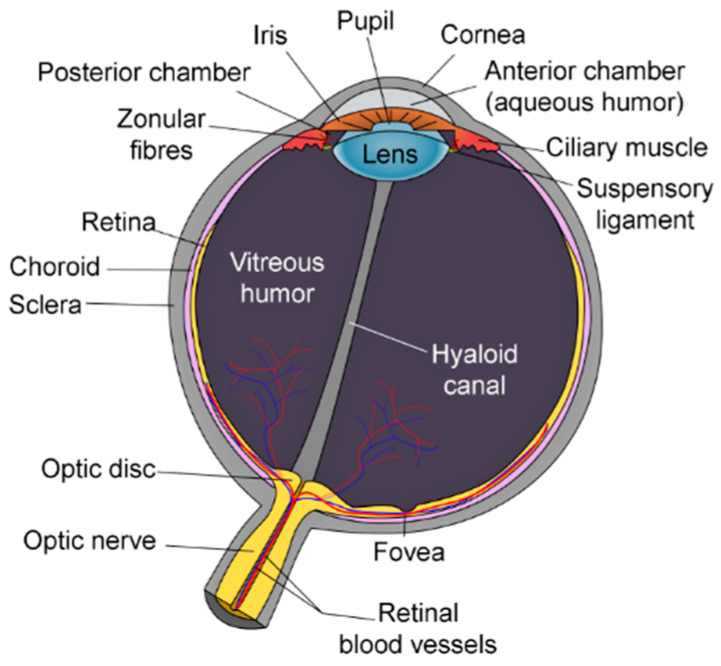
Human eye anatomy [3].

The human eye is a complex organ consisting of three main layers: The outermost layer is composed of the cornea, sclera and conjunctiva, the middle one consists of the choroid, ciliary body and the iris, and the innermost retina. Within these structures, there are vitreous body, crystalline lens, and aqueous humor, etc. The basic human eye anatomy is depicted in Figure 1 [1,2,3].

The anterior segment of human eyes mainly includes the cornea, corneo-limbal junction, sclera, anterior chamber, posterior chamber, iris, ciliary body, and lens [4]. The cornea has a diameter of about 11.5 mm and a varying thickness from 0.5 mm in the center to 0.8 mm at the periphery, contributing nearly two-thirds of the total refractive power and plays an important role in the optical performance of the eye [4,5,6]. The dimension of the anterior eye surface encompassing the corneo-scleral area is up to 20 mm in diameter [7,8,9].

The geometric shapes of the anterior segment have received increasing attention from researchers in various fields. In the clinical practice of ophthalmology, the geometric characterizations of the anterior segment have become a crucial analysis due to the multiple applications of the analysis on the anterior segment structures for optimizing corneal and intraocular refractive surgery procedures [10,11,12]. The shapes of the anterior segment are essential to the three-dimensional computer modeling of the human eye, especially to the personal eye modeling [13,14], the binocular eye modeling [15], and eye modeling under accommodation [16,17]. For optimum performance and ocular health, the contact lens and intraocular lens design require specific fitting principles to be adhered to. To assist with the fitting procedure, a comprehensive understanding of the shape profiles and positions of corneal, scleral, and lens in the human eye is necessary [18,19,20].

**Figure 2:**
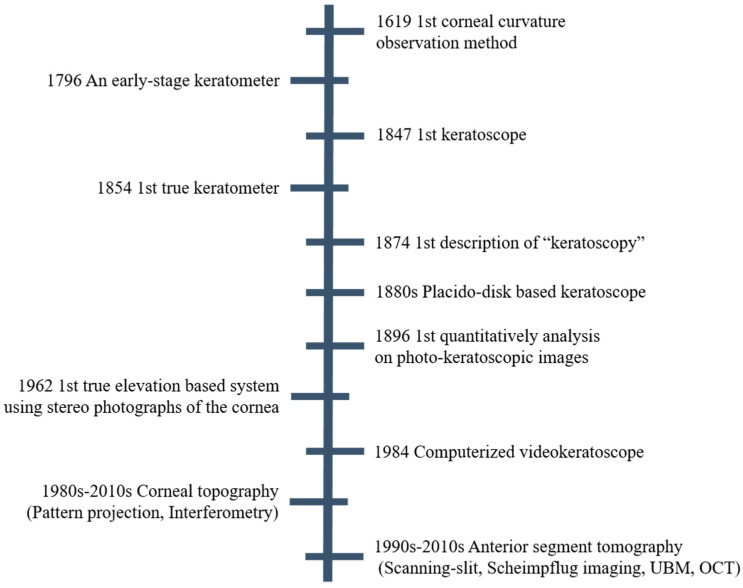
Development of the anterior segment measurement technologies.

Various technologies have been invented and innovated in the historical development of the anterior segment measurement, as shown in Figure 2.

In 1619, Scheiner discovered a qualitative observation method for the corneal curvature by matching the size of the image reflected from the cornea with that produced by one sphere with a known radius [21]. In 1796, Ramsden et al. developed an instrument to measure the corneal curvature, which is considered as an early-stage keratometer [22,23]. The first true keratometer was developed by Van Helmholtz in 1854. The instrument was later improved by Javal, Schiotz, and other researchers [23]. The corneal curvature and power can be calculated by these keratometers using the relationship among the object size, image size, and object distance. However, the keratometers can only measure a small region of the cornea (two points at the central 3–4 mm zone) [24]. Additionally, the corneal curvature was roughly measured by considering the cornea as a simple convex mirror, assuming the regularity of curvature over the corneal cap. Thus, limited quantitative information can be provided [24].

Measuring the entire corneal shape has become more important in clinical practice. Accurate measurement on corneal topography including the central and peripheral zones is crucial to determining the quality of vision, detection and diagnosis of pathology, prescription of noninvasive and invasive treatments, and evaluation of therapy efficacy [25,26]. This led to the development of keratoscopy. Keratoscopy can be employed to evaluate about 60% of the total corneal area which is the major advantage over keratometry. The first keratoscope was invented by Goode in 1847, using the reflection of a square object from the cornea from the side of the target [27,28]. In the same year, Cuignet established the first description of the technique “keratoscopy” [28,29]. In addition to these pioneer works, Placido invented the Placido disk to measure the corneal curvature by photographing the reflections on the anterior corneal surface of a series of illuminated concentric rings in the 1880s, which is still currently being used [30]. In 1984, Klyce introduced a new computer-based videokeratoscope that transformed the gross keratoscopic examination of the cornea into the era of computerized corneal surface analysis [31]. Since then, videokeratoscopy has become an increasingly important tool for assessing the anterior corneal curvature, the patterns of power distribution, and the degree of corneal irregularity [32,33], also known as the early-stage “topography”. However, the data captured from the peripheral cornea are not accurate, and the mires of most of the systems exclude central and paralimbal areas. Most importantly, this technique is not able to image the posterior surface of the cornea [34]. Unlike traditional techniques discussed previously, comprehensive topography means to measure the X, Y, and Z coordinates of the surfaces directly. Using pattern projection and interferometry techniques, corneal topography processes the pattern of light rays reflected off the cornea to reconstruct the anterior corneal surface.

The importance of the corneal thickness measurement was then realized in diagnosis of several corneal disorders, including corneal degenerations, endothelial dysfunction, and different types of stromal dystrophy [35]. Additionally, the high-resolution thickness measurement of the cornea and lens was heeded due to the development of the corneal refractive surgery. To solve that issue, several techniques were developed to obtain information from both anterior and posterior corneal surfaces, called the “tomography”. In contrast to corneal topography, anterior segment tomography not only obtains quantitative information from both the anterior and posterior corneal surfaces, but also has the capability of imaging the anterior segment tissues and reconstructing the 3D shapes of the tissues digitally [31,36]. Based on different optical or ultrasonic principles, the technologies for anterior segment tomography have been developed rapidly in the past two decades. All of these technologies aim to achieve accurate evaluation of the geometric characteristics of the anterior segment, including the curvature, asphericity, thickness of the cornea and lens, as well as the dimensions of the anterior segment space, etc.

The technologies for measuring the geometric shapes of the cornea or the anterior segment evolved from qualitative analysis to quantitative measurement. This paper reviews the principles of the corneal topography and anterior segment tomography technologies. The advantages and limitations of each technology are presented respectively, along with their applications. The future trends of technical development are outlined at last.

## 2. Approaches in Measuring the Anterior Segment

There are various available technologies for measuring the anterior segment at present, including pattern projection, interferometry, Scanning-slit, Scheimpflug imaging, very high frequency digital ultrasound or ultrasound biomicroscopy (UBM) and optical coherence tomography (OCT) [21]. Several commercial instruments are multifunction systems that combine the Placido disk corneal topography with the tomography technologies to increase the accuracy of the anterior corneal curvature measurement [26,37,38], which are described in the following sections.

### 2.1. Pattern Projection

#### 2.1.1. Placido Disk

**Figure 3:**
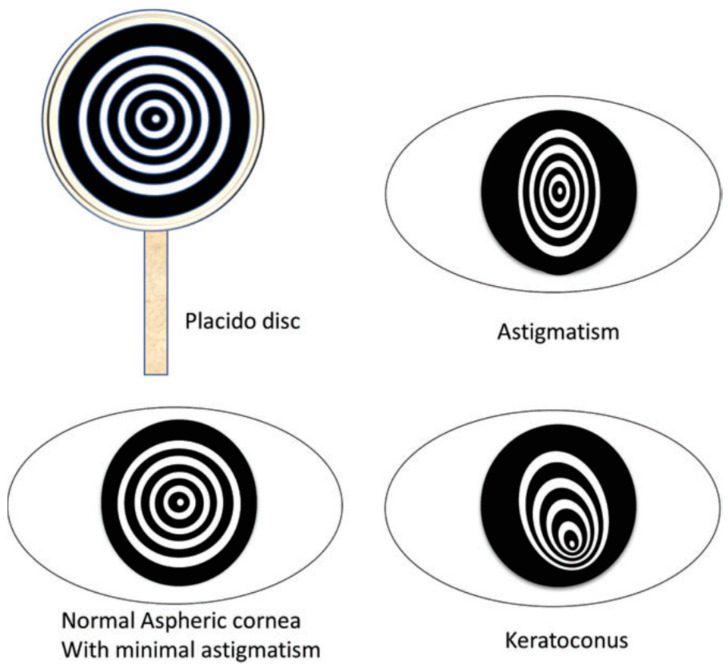
Placido disc and representative patterns of corneal shapes [36].

Placido disk is a device made of concentric rings with different color, typically white rings on a black background, as shown in Figure 3 [39]. The shape of the anterior corneal surface can be inferred by inspection of the light of the projected concentric rings reflected on the cornea. The rings follow lines of the equal slope which is like a topographic map of a mountain. The reflective rings appear closer together on the steeper parts of the cornea and farther apart in flatter areas. There are several algorithms to process the image of the Placido disk’s rings and to reconstruct the corneal surface by tracing radial lines that all pass through the center of the rings in the image [40,41].

In 1896, Gullstrand [30] photographed the reflected corneal image of coplanar rings to measure the central part of the cornea. To evaluate a larger region of the corneal surface, Knoll et al. [42] introduced a hemispherical surface for the rings which made the analysis of the image of the rings simpler [43].

The significant advantage of the Placido disk-based technology is its simplicity. However, the Placido disk systems also present some limitations [44,45]. All the rings must be in focus for accurate data acquisition, while this becomes more difficult in the peripheral cornea and thus limits the survey area [44]. Some minor and insignificant variations are present in every cornea but may not be detectable on the inspection of a Placido disk image. The measurement resolution is thus limited. Moreover, the Purkinje images may confuse the analysis of the rings [5]. Errors may occur in the surface reconstruction when the cornea is irregular. Once the rings merge or cross with each other due to poor surface quality, the reconstruction may be failed. Additionally, the posterior corneal surface cannot be investigated. Therefore, the total corneal power calculation is compromised by using the assumption of a constant ratio between anterior and posterior corneal surface radii of curvature [46] which is in fact not constant [47]. Nowadays, many hybrid systems are reinforced by combining the Placido disk with other technologies such as Scanning-slit and Scheimpflug imaging [36].

#### 2.1.2. Rasterstereography

**Figure 4:**
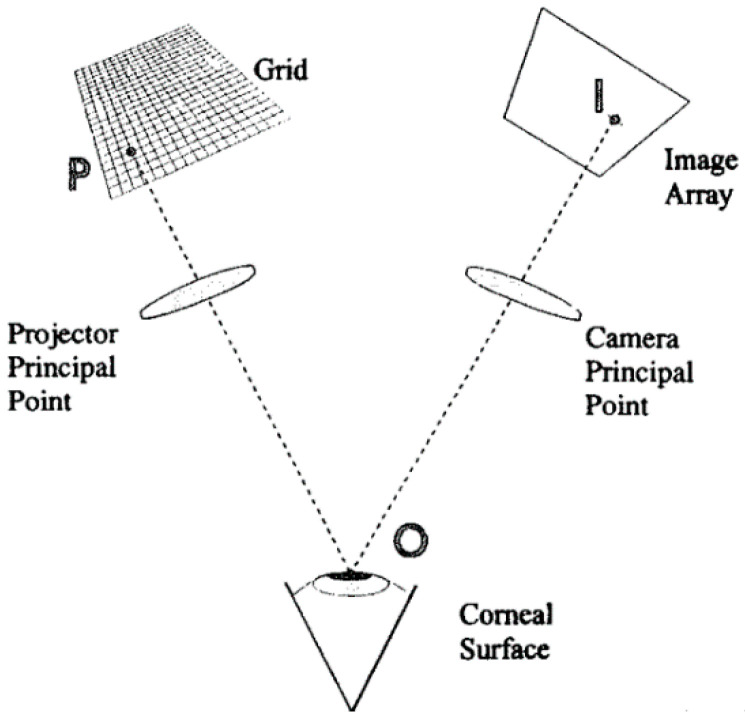
Triangulation principle for corneal surface measurement [48].

In the 1980s, the PAR corneal topography system (PAR CTS) mentioned previously projected a raster pattern (composed of horizontal and vertical lines spaced about 0.2 mm apart) onto the anterior corneal surface [48]. Based on the rasterstereography (stereo triangulation principle), the X, Y, and Z coordinates of the anterior corneal surface can be directly computed point-by-point, as shown in Figure 4 [49]. Although the system is no longer commercially available, it is widely recognized as the first system to measure the elevation data [39].

Rasterstereography can be adapted to measure over 12 mm on the corneal surface with the accuracy of several tens of micrometers [23]. Rasterstereography requires neither a smooth reflective surface nor precise spatial alignment for accurate imaging of the corneal surface. However, the sensitivity and accuracy depend on the angle between the axes of the projector and imaging system which determines the distance between two consecutive projected fringes on the corneal surface proportionally [43]. Furthermore, analogous to the Placido disk, the surface reconstruction of the irregular cornea fails due to the fact that the raster pattern could merge or cross with each other.

### 2.2. Interferometry

In addition to pattern projection, several interferometry approaches have been applied to characterize the corneal profiles. When two coherent and monochromatic wavefronts are superimposed after traveling paths with different length, the interference fringes would be formed. The fringes contain both the amplitude and phase information, which can be applied for 3D reconstruction. This is the basic phenomenon of interferometry.

#### 2.2.1. Holographic Technique

**Figure 5:**
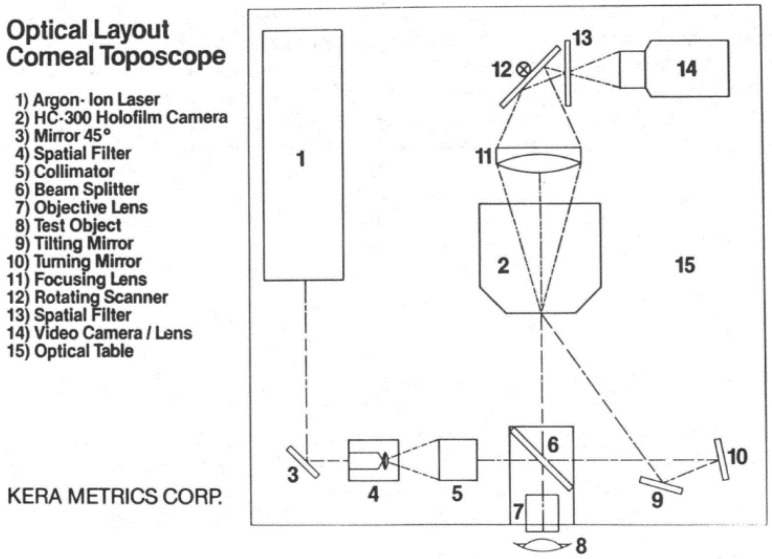
Two-wavelength holographic interferometer for contour evaluation of human corneas [53].

Theoretically, the holographic technique can obtain sub-micrometer sensitivity to height variations by using two mutually coherent beams and recording the interference pattern generated on the corneal surface by two coherent wavefronts, as shown in Figure 5 [50,51,52].

In 1966, Grolman and Lawton produced a hologram of the human eye for the first time and predicted its potential applications in ophthalmology [54,55]. In 1972, Calkins pointed out the potentials of holography in the examination of the corneal elasticity in vivo and showed that the heart beat itself can cause a fine interferometric fringe pattern in the corneal image [56]. In 1977, Poltich [57] introduced a method to obtain contour fringes from a living human cornea by applying a two-wavelength technique. Using laser holographic interferometry fringe patterns, a system CLAS 1000 was released to depict the corneal profiles [58,59] though the device is not commercially available now.

The holographic technique facilitates interferometry as it realizes full viewing field and real-time measurement without high requirements on the optical components quality (lenses and mirrors). The equipment is rather complex by using an analogous interferometric system to maintain the correct positions of the optical components. Moreover, the hologram is particularly easy to be affected by the vibrations and air turbulence. It is very hard to ensure the measurement stability [53,60].

#### 2.2.2. Moiré Technique

**Figure 6:**
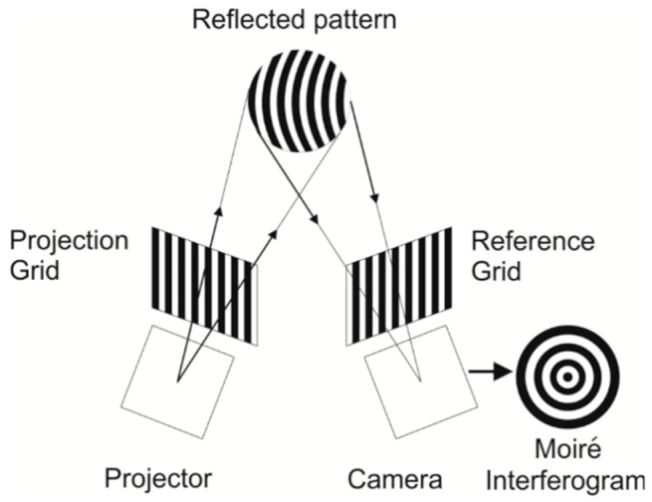
Schematic diagram of the projection Moiré profilometry [61].

Moiré fringes occur when two sets of parallel lines are superimposed at different orientations such as displaced, rotated, or having a slightly different pitch. A grating called “active grating” is projected on the corneal surface. The active grating deforms with the corneal profile. The deformed and reflected grating from the anterior corneal surface is re-imaged on a reference grating to produce the Moiré fringes which represent the contours of equal phase difference correlating to height contours, as shown in Figure 6. Then, the direct corneal topographic information can be obtained.

**Figure 7:**
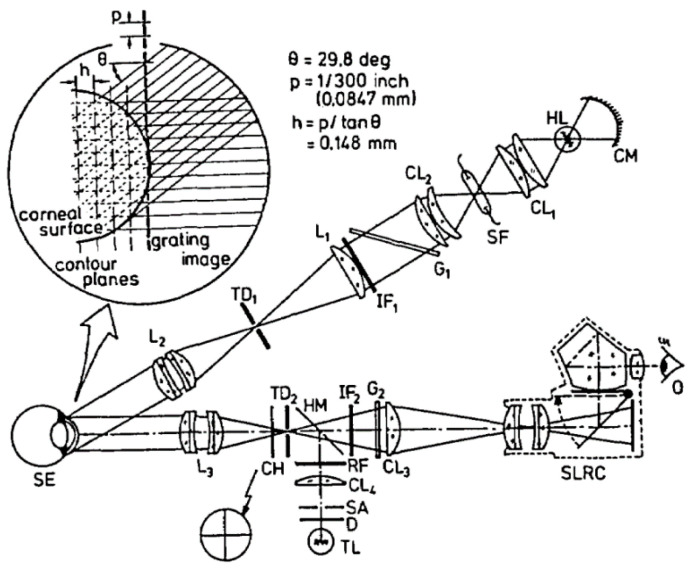
Schematic diagram of Kawara’s design [63].

**Figure 8:**
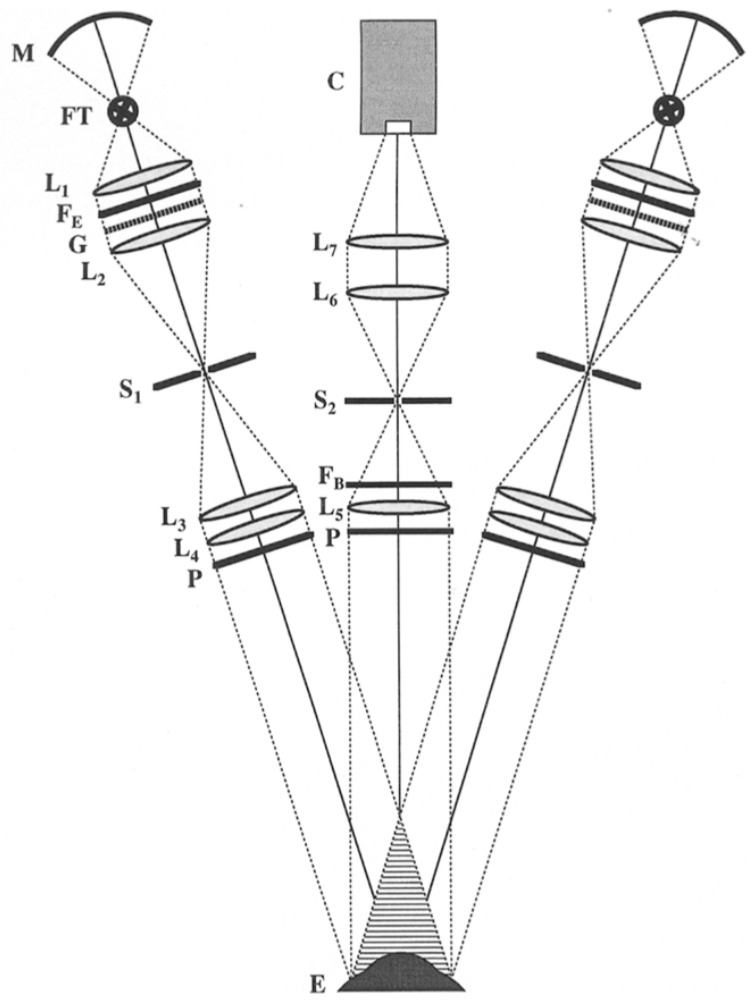
Optical schematic of the Maastricht Topographer [65].

**Figure 9:**
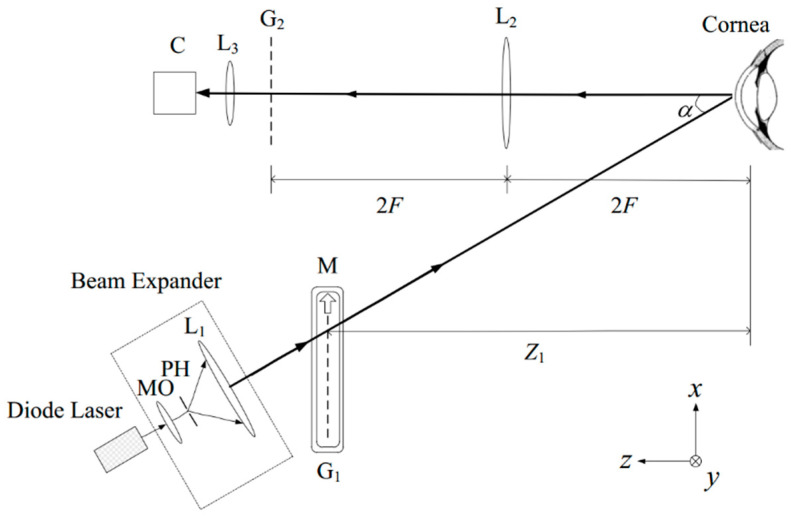
Setup for measuring the corneal surface based on the Moiré method [67].

In the 1960s, Mandell [62] described a technique using Moiré patterns to measure the corneal curvature. In 1979, Kawara [63] projected sine wave gratings by the telecentric system to generate the Moiré fringes and obtained the height profile of the corneal surface. In 1987, Adachi et al. [64] developed a device to measure the corneal as well as the limbal contour based on the Moiré technique. In 1995, referring to Kawara’s design shown in Figure 7, Maastricht Shape Topographer shown in Figure 8 was developed to measure the corneal surface elevation point-by-point based on the projection of two sinusoidal gratings and the analysis of the distortion of the gratings caused by the corneal-scleral shape [65,66]. More recently, in 2013, a method for reconstructing the corneal surface profile based on the Moiré method and heterodyne interferometry using a simple optical setup was described in Figure 9 [67,68].

The significant advantages of the Moiré technique include no need for mathematical assumptions on the corneal shape and equal accuracy in the center and the periphery. To guarantee the measurement accuracy, the fringe pattern needs to be clearly distinct. To achieve high resolution, very dense fringes have to be used which may cause the concentration of the fringes at the region with a large gradient.

#### 2.2.3. Twyman-Green Interferometer

**Figure 10:**
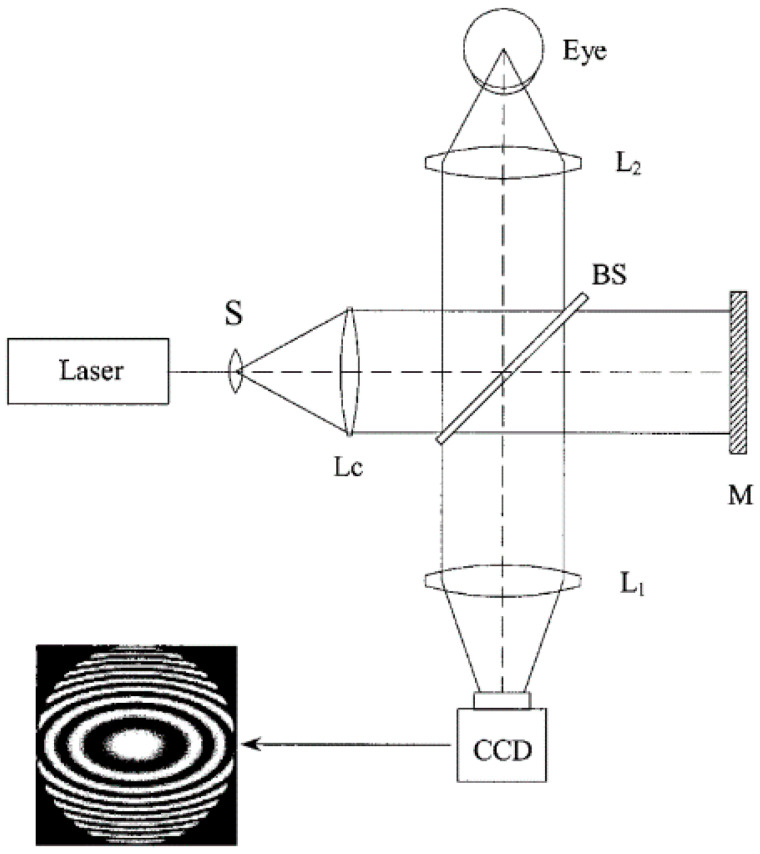
Twyman-Green interferometer for testing the corneal surface [43].

Twyman-Green interferometer is a powerful tool to measure the surface of the optical elements such as mirrors and lenses. The cornea can act as a convex mirror in a Twyman-Green interferometer, as shown in Figure 10 [43].

In 1990, Risaliti and Ronchi [69] proposed a Twyman-Green interferometer (TGI) which projects a collimated beam on the corneal surface and produces the interference pattern by the superposition between the reflected beam and a spherical divergent reference beam. In 1994, Hochberg and Baroth [70] used a TGI with a white light source to produce a polychromatic interference pattern on the anterior corneal surface and estimated the local height by spectral analysis. In 1995, Kasprzak et al. used a TGI with a He-Ne laser to obtain real-time subtraction interferograms from the human cornea in vivo with a best-fit sphere limited to a region of 5 mm in diameter. [56] Rottenkolber and Podbielska [71] designed a laser TGI with a piezo translator to achieve high precision measurement of ophthalmic surfaces based on the phase shift technique in 1996. In 1999, Licznerski and Kasprzak [72] proposed a technique of corneal topography using a double path Mach-Zehnder interferometer called the lateral-shearing interferometer. In 2001, Kowalik et al. [73] used a radial shearing interferometer based on the Mach-Zehnder system to produce the interferograms of the cornea. In 2002, Kasprzak and Jaroński [74] presented a technique for in vivo measurements of dynamic variations of the corneal topography by use of the TGI. In 2005, Licznerski et al. [75] developed a double path shearing interferometer for corneal topography measurement. In 2016, Micali and Greivenkamp [76] designed a dual interferometer system for measuring the dynamic corneal topography using two aligned simultaneous phase-shifting polarization-splitting TGIs.

#### 2.2.4. Fourier Transform Profilometry

Fourier transform profilometry (FTP) is another interferometry approach needing only one (or two) fringe(s), and allows full-field analysis and high precision. By projecting a Ronchi or sinusoidal grating onto the surface, the height information is encoded into a deformed fringe pattern. The surface shape can be decoded by calculating Fourier transformation, filtering in the spatial frequency domain, and calculating inverse Fourier transformation [77,78].

**Figure 11:**
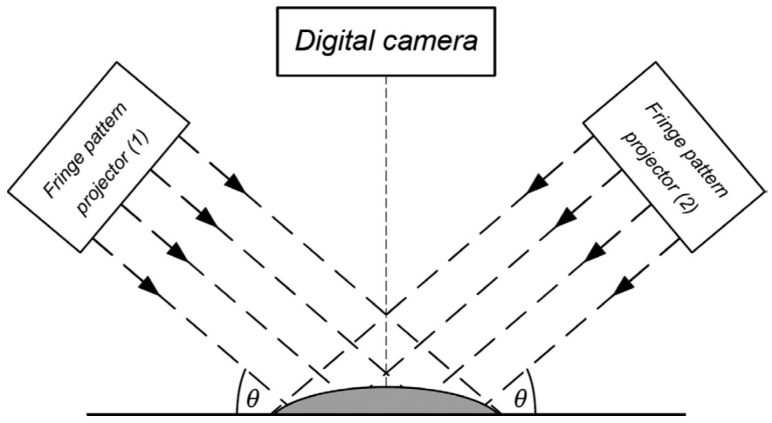
Eye surface profiler using two symmetrical projectors [80].

In 2002, Klein et al. [79] introduced a prototype of a height-based topographer (Euclid System) projecting about 40 slits onto the cornea simultaneously which can cover the surface up to 16 mm vertical and 20 mm horizontal. The Euclid system uses the Fourier transform method for calculating the phase shift, from which the height can be calculated. In 2012, Iskander used Fourier based image processing and phase retrieval algorithms to achieve successful measurement of anterior eye surface topography. Then, Iskander et al. presented a methodology of anterior eye surface measurement based on advanced Fourier transform profilometry and presented the first accuracy and precision results for the eye surface profiler (ESP) in 2015 [80]. The ESP is capable of determining the curvature and elevation up to 20 mm diameter, which covers the cornea, corneo-limbal junction, and sclera by using a double projection Fourier transform profilometer [7], as shown in Figure 11.

The interferometry technologies are capable of measuring the elevation of the anterior corneal surface directly with higher accuracy than the traditional Placido disk-based system [23]. These technologies can be employed to achieve the accuracy of several micrometers but only cover the corneal region [43]. Moreover, it is imperative to instill fluorescein to the tear film as a diffusing agent for distinctly imaging the anterior corneal shape [67,76,80]. Additionally, the major drawback is that the low-pass filter should be inevitably applied during the interference fringe processing. It cuts off or smooths out the high frequency components in the image, such as sharp, stepped, and fine structures on the cornea [81]. The resolution is thus limited.

### 2.3. Scanning-Slit

The Scanning-slit technique was applied in the first elevation system with the capability to measure both the anterior and posterior corneal surfaces [21]. Based on the light scattered from the transparent corneal structures and the optical triangulation concept, the Scanning-slit technique made a further improvement in corneal measurement to define the spatial relationship between the anterior and posterior corneal surfaces and reconstruct the cornea in three dimensions [31,82].

**Figure 12:**
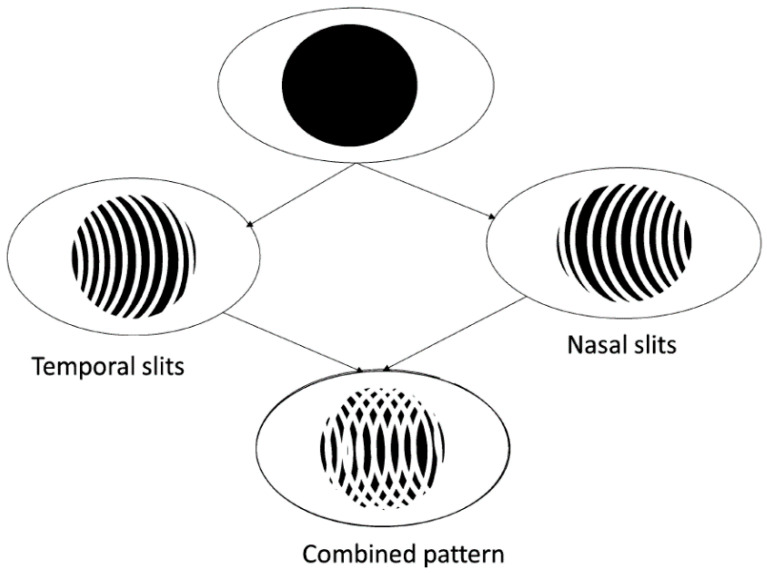
Overlapping slits to map the cornea using the Scanning-slit technology [36].

Orbscan I was the first commercial Scanning-slit system launched in 1995 [34], in which a slit lamp projects a beam at 45° onto the cornea. Twenty slits are projected sequentially on the eye from the left and then from the right, as shown in Figure 12. Then, a total of 40 slits are acquired using a video camera at the pre-specified positions in two 0.7 s periods, which produce 240 data points per slit [34,83]. Based on the optical triangulation concept, the pixel points per slit on each image are calculated to generate the 3D corneal profiles, corneal power, and pachymetry maps [49]. Later, the Placido disk system was added in the second version of the device Orbscan II/IIz which can map the corneal surface of 11 mm in diameter [84]. The Orbscan systems utilized parallel segmental cross-sectioning (no shared points) and relied on the Placido disk image to assist in the image registration for the 3D reconstruction [23]. The anterior elevation was calculated with the help of the Placido disk system but the posterior elevation was extrapolated through keeping the curvature ratio of anterior and posterior surface to be constant. Thus, the system was prone to error in the post-refractive surgery eyes [34,82]. As the key technology of the Scanning-slit system, the edge-detection algorithm for the slit beam stripe is vulnerable to interference from the corneal reshaping after clinical surgery [84]. The measurement with this device is significantly dependent on many factors, such as inevitable eye movement, the stability of tear film, corneal transparency, and the presence of corneal abnormalities.

Numerous researches and articles have highlighted certain problems with the Scanning-slit system particularly in identifying the posterior corneal surface and the underestimation of pachymetry after refractive surgery. Due to the non-planar shape of the cornea, the data in the peripheral region acquired by the centrally located Scanning-slit based camera are unreliable [36]. The depth of focus of the technique is limited. Hence, the assessment of the posterior lens is not possible [85]. On the other hand, the back-scattered light from the limbal or scleral region is too strong so the camera is driven to saturation. It is thus impossible for this technique to measure precisely in these regions [79]. The speed, accuracy, and reproducibility of the Scanning-slit technology are insufficient in comparison with other technologies such as Scheimpflug imaging [86,87,88].

### 2.4. Scheimpflug Imaging

**Figure 13:**
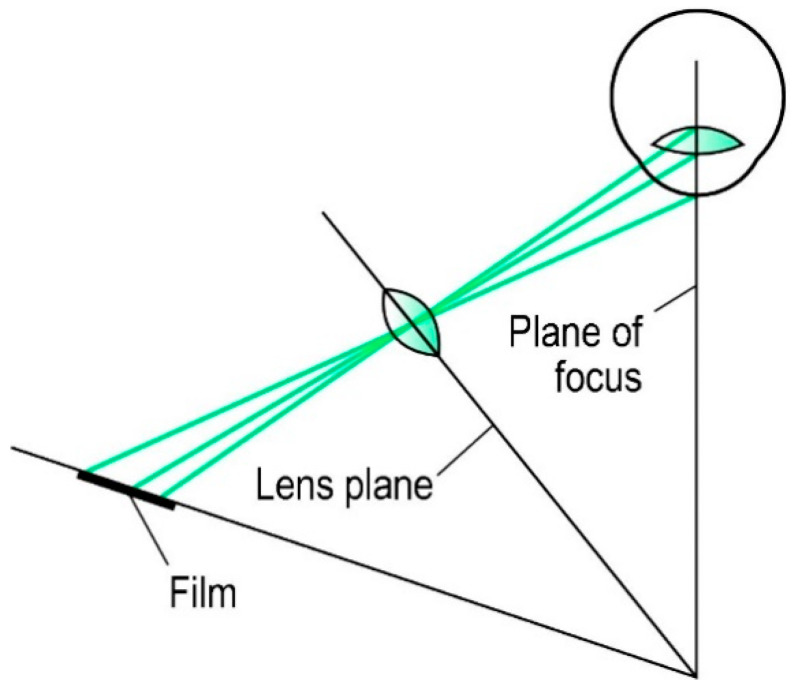
Principle of Scheimpflug imaging [90].

The non-planar shape of the cornea can potentially lead to spurious results in Scanning-slit systems as mentioned above. Therefore, the Scheimpflug principle was applied in corneal imaging as an alternative technique that can measure the posterior corneal surface using the light scattering and optical triangulation concept [89]. The Scheimpflug principle, first introduced at the beginning of the twentieth century, describes an optical imaging condition: When a planar subject is not parallel to the image plane, an oblique tangent can be drawn from the image, object, and lens planes. By tilting an angle of the image plane with respect to the optical axis carefully, the increased depth of focus and minimal image distortion can be achieved. In this case, the Scheimpflug imaging system can provide cross-sectional images of the whole anterior segment with larger depth of focus and better spatial accuracy than the Scanning-slit system commonly using a traditional camera containing a coaxial optical path, as shown in Figure 13 [23].

The rotating Scheimpflug imaging systems project a narrow slit beam approximately perpendicular to the corneal surfaces, scan the anterior segment rotationally, and capture the scattered light [91]. As a precise and versatile tool for anterior segment imaging, rotating Scheimpflug imaging systems enable the measurement of corneal shape (anterior and posterior corneal surfaces) [92,93,94], pachymetry from limbus to limbus [95,96,97], anterior chamber depth [98], anterior chamber volume [91], curvature and elevation of the crystalline lens surfaces [99,100], etc.

The first Scheimpflug imaging system was set up by Brown for the investigation of human accommodation and presbyopia in the 1970s [101]. The first rotating Scheimpflug camera using black-and-white film was developed by Dragomirescu and Hockwin which was later turned into a commercial Scheimpflug camera called Topcon SL-45 [102,103]. An Oxford group introduced a nonrotating Scheimpflug camera marketed as Oxford CASE 2000 [104]. The first rotating video Scheimpflug system was released as Zeiss SLC. Sasaki et al. [105] designed the first electronic rotating Scheimpflug camera and marketed it as EAS 1000. Nowadays, the Pentacam is one of the most recognized Scheimpflug imaging systems which uses a rotating Scheimpflug camera to obtain 50 Scheimpflug images of the anterior segment in less than 2 s [106,107,108]. Then, Oculus presented upgraded versions of the Pentacam, the Pentacam HR and AXL [109]. Other systems based on the Scheimpflug imaging technique released commercially are the Galilei, SIRIUS, and TMS-5. The Galilei and the SIRIUS are both hybrid systems integrating a Placido disk system [89].

The rotating Scheimpflug imaging systems offer certain advantages over the scanning-slit systems. One major advantage is the more accurate representation of the posterior corneal surface individually to make the measurement of pachymetry more precise both in the center and in the periphery, especially in postoperative eyes where the curvature ratio between the anterior and posterior surfaces is altered [34,110]. Another one is that the images acquired by Scheimpflug imaging systems share a common point (center of rotation) which makes the image registration much easier and more accurate to construct the three dimensional model of the anterior segment [92].

The Scheimpflug imaging technique also has several limitations, such as geometrical distortion and optical distortion. Consequently, correcting the images for the distortions is crucial in all Scheimpflug applications to obtain reliable geometrical information on the cornea, anterior chamber, and intraocular lens, etc. [111,112]. The Scheimpflug camera and light source need to be aligned accurately to ensure repeatability for a given eye [91]. The tear film stability and eye movement influence the accuracy of the measurement due to a period of time required to complete rotation during the image acquisition [45]. Similar to the Scanning-slit technique, the back-scattered light is oversaturated so that the image quality of the opaque tissues (such as the limbus and sclera) is poor [113]. The Scheimpflug imaging technique cannot visualize the entire lens and anterior chamber because of the pupil aperture, which can be precisely observed by ultrasound biomicroscopy (UBM) and OCT [114,115,116,117]. Additionally, the resolution of the images is lower than that of OCT [85].

Different methods have been applied to correct the optical distortion of the Scheimpflug imaging technique. Ray-tracing techniques were applied to obtain reliable surface geometry from Scheimpflug images [23]. A method based on Hough transform was proposed in order to obtain corrected surfaces from Scheimpflug images [118]. Scheimpflug imaging also have been thoroughly validated and corrected from the optical distortions produced by the preceding ocular surfaces and the accuracy of the results was demonstrated using model eyes with known geometry [119].

### 2.5. Ultrasound Biomicroscopy

Although non-contact optical methods are precise, cost-effective, and easy to use, optically nontransparent structures including normal anatomy (sclera, iris) and pathology (haemorrhage, corneal scars, cataract) are hard to be imaged clearly and distinctly using optical methods. By contrast, ultrasound technology can visualize the anterior segment structures in vivo at microscopic resolution even in the presence of optical opacities [120,121,122].

**Figure 14:**
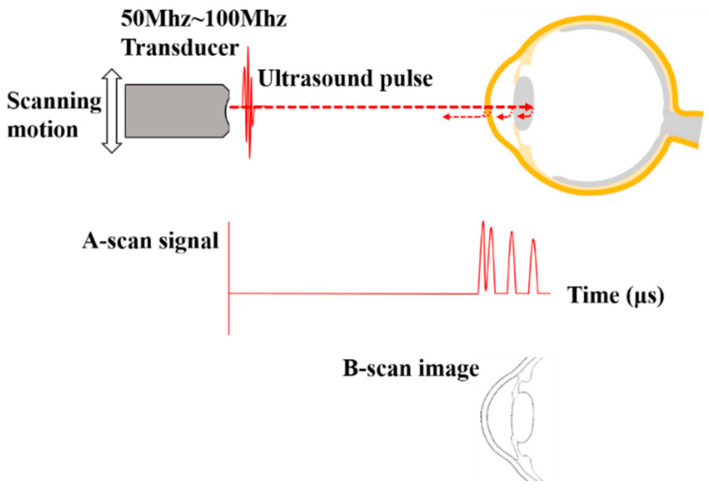
The basic principle of ultrasound biomicroscopy (UBM). The transducer generates an ultrasound pulse. The pulse encounters the anterior segment tissues. The backscattered echoes would be detected by the same transducer, resulting in the A-scan signal to indicate the distances between the tissues. The B-scan image of the anterior segment could be obtained by scanning.

Analogous to optical biomicroscopy, the term “ultrasound biomicroscopy” is preferable to describe the ultrasonic visualization of the living tissue than other terms as “ultrasound backscatter microscopy” or “high-frequency ultrasound” or “very high-frequency ultrasound” in the literature [123]. UBM is qualified in both qualitative and quantitative assessments of the anterior segment. It uses a transducer to generate high frequency ultrasound pulses, as well as gathers the homologous back-scattering echo signal from ocular tissues with differing sonic refractive index. The time delay between the emission and echo of each pulse can be converted into image information, as shown in Figure 14 [111,124]. Such advances in the transducer, high-frequency signal processing, and precise motion control technology promote the development of UBM [125].

Instead of using traditional 10 MHz ultrasound frequency for the whole eye imaging with an approximately 150 µm resolution, UBM uses ultrasound frequencies in the range of 50 to 100 MHz range to provide real-time cross-sectional images with the penetration depth of 4–5 mm and the resolution of 20–50 μm [45,126]. UBM is able to image not only the whole anterior segment, including the conjunctiva, scleral, cornea, anterior chamber, iris, ciliary body and lens, but also the vitreous humour and retina [120,121,122].

The original application of ultrasound for imaging the eye can be traced back in the 1950s with the work of Mundt et al. [127] (A-scan) and Baum et al. [128] (B-scan). The first practical UBM system using the 50 MHz probe to image the entire anterior segment in B-scan mode was developed by Foster et al. in the early 1990s [129,130,131,132]. The first commercial instrument consisted of a 50 MHz probe, and it was supported by an articulated arm. Numerous manufacturers produce UBM systems with higher scan rates, more compact and stable handheld probes than that of the original ones. Some of these instruments provide a scan width sufficient to encompass the entire anterior segment [120].

As mentioned previously, the superiority of UBM is that ultrasound can penetrate through opaque tissues. Although commonly being used to compare with the other technologies, UBM has a lot of disadvantages which makes it a terrible standard [23]. In UBM, using higher ultrasound frequency, the image resolution improves, while the trade-off is the poorer penetration. The attenuation of ultrasonic waves also increases with frequency and thus limits the penetration and imaging quality [85]. Since most energy of the ultrasonic waves is scattered over a wide range of angles at small tissue structures, only a small fraction of the ultrasound can be reflected. This effect makes these tissue structures hard to be visualized clearly. Moreover, UBM is a contact technique which can be achieved through immersion, requiring physical contact between the cornea and the probe using a coupling medium. In some cases, a saline-filled eyecup is placed at the cornea in order to modify the anatomical dimensions or the pressure of the anterior segment [120]. Additionally, the refractive index of the acoustic coupling medium is a little different from that of the cornea which may cause inaccuracies of curvature. Moreover, the accuracy of the measurement using UBM is sensitive to probe positioning and angulation. Thus, UBM requires an experienced operator and topical anesthesia to avoid possible corneal abrasion and inevitable eye movement [111]. The contact and time-consuming nature of the examination limit the application of UBM [114].

### 2.6. Optical Coherence Tomography

Optical coherence tomography (OCT) is a non-invasive and non-contact optical imaging technique for visualizing the internal morphology of the human eye in vivo [133,134,135]. It can provide detailed, magnified cross-sectional images of the ocular structures with micron-scale resolution and hundreds of KHz or even MHz scan rates [136,137,138]. Compared to UBM, this technique utilizes the emission and reflection of light instead of sound [139,140], generating two or three dimensional tomographic images of the cornea and other anterior segment tissues by measuring the time-delay of the reflected or backscattered light from the ocular structures with different reflectivity [141,142,143]. Since the speed of light is much higher than that of sound, the measurement of echo time delay requires a temporal resolution of the order of femtoseconds which cannot be performed directly at present [85,144]. Thus, OCT employs low-coherence interferometry (LCI) to extract echoes of light and to find the optical distances from the interferometric fringes [36,145]. Based on LCI, OCT can achieve axial resolutions from 3 to 20 μm [89,140] by comparing a partially coherent reference beam to the ones reflected or backscattered from the specific layers in the anterior segment. Nowadays, OCT provides high sensitivity, large dynamic range and micron level resolution imaging approach for the cornea, limbus, anterior scleral contour, apical and limbal clearance, anterior chamber, and some other ocular structures beyond the cornea. In addition, contact lens fitting and intraocular lens (IOL) power calculation can be implemented using OCT data [146,147].

In general, OCT technologies use a near-infrared light source (the central wavelength is among 0.8–1.55 μm) or a broadband light source, such as super luminescent diodes, super bright LEDs, femtosecond pulse lasers, and incoherent white light source, etc. [144]. The most common configuration of OCT technologies is the Michelson-type interferometer, in which the light source is split into a reference beam and a sample beam [148]. The reference beam is focused onto the mirror in the reference arm. The sample beam is directed to the tissue and then scattered back. The interference fringes to be recorded as a depth profile (A-Scan) can only occur when the two beams have traveled approximately the same distance within the coherence length of the light source [119]. Thus, using the near-infrared light source with longer single wavelength, OCT technologies can obtain shorter coherence length. In anterior segment OCT (AS-OCT) imaging [149], the light centered around 1300 nm wavelength can be less scattered in the intraocular tissues especially the opacities than the one centered around 800 nm wavelength. The higher wavelength leads to the deeper penetration. The light centered around 1300 nm wavelength is strongly absorbed by water in ocular media. Therefore, only 10% of the light can reach the retina which can improve the retinal protection and allow the use of high power illumination to enable high speed imaging [110]. By scanning the beam over the anterior segment in the longitudinal direction, the depth profiles (A-Scan signal) build up the B-Scan images [150].

During the development, OCT has experienced three main technical generations of revolution and evolution. The first generation OCT systems directly detecting the time delay of optical echoes was published in 1991, which is retrospectively referred to as time domain OCT (TD-OCT) [151]. Then, the alternative approach to TD-OCT named Fourier domain OCT (FD-OCT) had been further studied since 1995. Along with the progress, FD-OCT performs in two manners, originally as spectral domain OCT (SD-OCT) and then as swept source OCT (SS-OCT) [152]. The latest generation of OCT called full field OCT (FF-OCT) was first introduced in 2002. FF-OCT is capable of producing 2D en-face images directly without the implementation of scanning beams [85].

#### 2.6.1. Time Domain OCT

**Figure 15:**
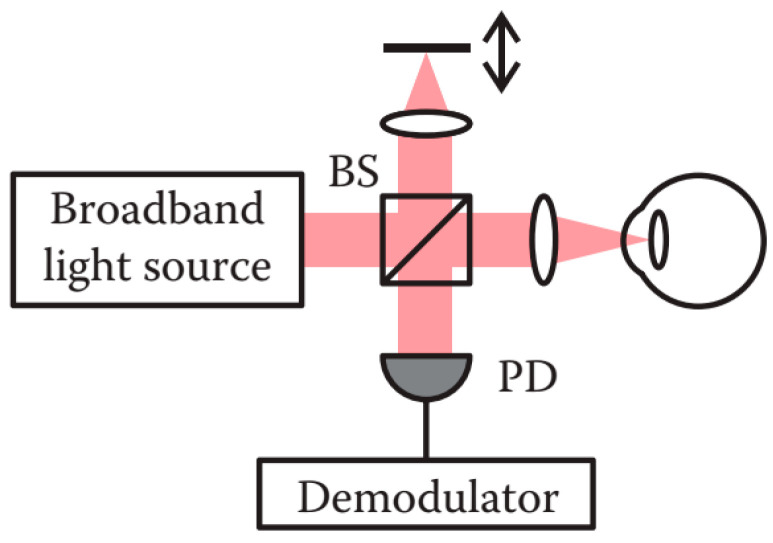
Schematic diagram of a time domain-optical coherence tomography (TD-OCT) system [85]. PD: Photodetector; BS: Beam splitter.

Implemented as Michelson-type interferometer with a broadband light source, TD-OCT systems detect the time-of-flight delay of optical echoes sequentially by mechanically generating a time-dependent depth scan of the interferometer reference arm. The reference arm is composed of an axial scanning mirror for A-Scan while the sample arm is configured with a lateral scanning system for B-Scan, as shown in Figure 15. The broadband point sensors such as photodiodes, i.e., are generally used in TD-OCT [137,153].

TD-OCT was first applied for retinal imaging in 1991 [151]. Anterior segment imaging using TD-OCT was first demonstrated in 1994 [149]. Then, commercial time domain anterior segment OCT (AS-OCT) systems were available, including the Visante AS-OCT, SL-OCT, Stratus OCT, and LENSTAR 900 adopting the rotating cube method to implement the scanning [154,155].

The notable disadvantages of TD-OCT are the low mechanical scanning speed of the reference arm and low detection sensitivity, resulting in long acquisition time. Additionally, it is difficult to speed up the system without compromising the signal-to-noise ratio. Consequently, TD-OCT can only achieve a sequential data acquisition rate of several thousand A-Scan lines per second, making it difficult to realize 3D scanning for in vivo human eye measurement [154,155,156].

#### 2.6.2. Fourier Domain OCT

To solve the problems in TD-OCT, the second generation called FD-OCT was developed, acquiring data in the spectral domain. In contrast to TD-OCT, the reference arm is fixed in FD-OCT [145]. Without mechanically modulating the reference mirror, the optical path length difference between sample and reference is encoded by the frequency of the interferometric fringes as a function of spectrum with all spectral components captured simultaneously [154]. The spectral interferogram was analyzed via fast Fourier transform to produce the A-Scan by extracting the position of intensity maxima. Thus, FD-OCT is capable of implementing continuously the acquisition of the spectral data and achieving dramatic increment in speed (tens of kHz vs. several kHz in TD-OCT) and sensitivity (20–30 dB higher than TD-OCT) [156,157,158].

FD-OCT was first demonstrated in 1995 [159]. Since 2002, FD-OCT has been introduced into corneal and anterior segment imaging [113]. Several companies, such as Carl Zeiss, Optovue, Heidelberg, Topcon, etc., have marketed different models of FD-OCT systems. FD-OCT can be further classified into two implementation strategies: Spectral domain OCT (SD-OCT) using a broadband light source with a spectrometer and swept source OCT (SS-OCT) using a frequency swept light source with high-speed detector [153,156,160].

##### Spectral Domain OCT

**Figure 16:**
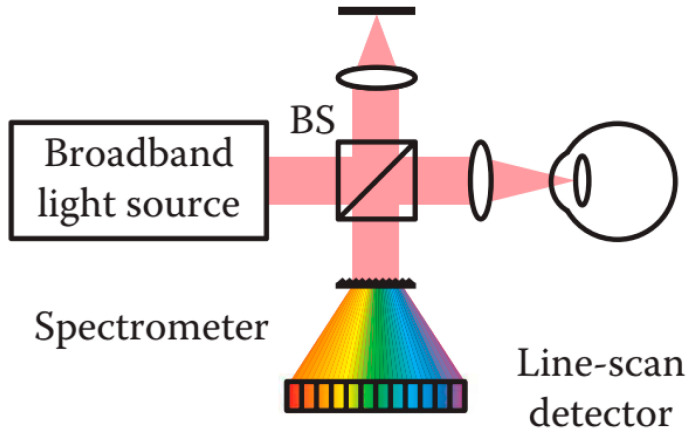
Schematic diagram of a spectral domain OCT (SD-OCT) system [85]. BS: Beam splitter.

SD-OCT systems utilize the Michelson interferometer configuration with a broadband light source and a fixed reference arm. Instead of using a photodetector in the case of TD-OCT, a high speed spectrometer is employed in SD-OCT to capture the interferometric signal intensity as a function of wavelength or frequency in the spectral domain [145]. A single A-Scan is obtained in one shot by the spectrometer with line-scan CCD or CMOS camera. The depth information is transformed into spectral intensity distributions in the pixels of the camera corresponding to a different wavelength or frequency, in which the spectral information is encoded in space, as shown in Figure 16 [137]. The conversion between wavelengths or frequencies and intensity in pixels is implemented by means of a Fourier transform to produce the depth profile. Since the entire A-Scan signal is measured simultaneously, a significant detection efficiency can be achieved.

The main drawback of SD-OCT is the strong SNR roll-off in depth. Moreover, the detection resolution of the spectral distribution is limited by the pixel size of the camera in the spectrometer. Thus, the echoed signal with the finer wavelengths or higher frequencies from the region of larger curvature in the cornea could be washed out [85,134]. Despite many studies on multiple-channel, multiple-focus and Optical Frequency Comb based approaches offering possible solutions to the problem [134,161], SS-OCT was developed to overcome the main drawback in SD-OCT.

##### Swept Source OCT

**Figure 17:**
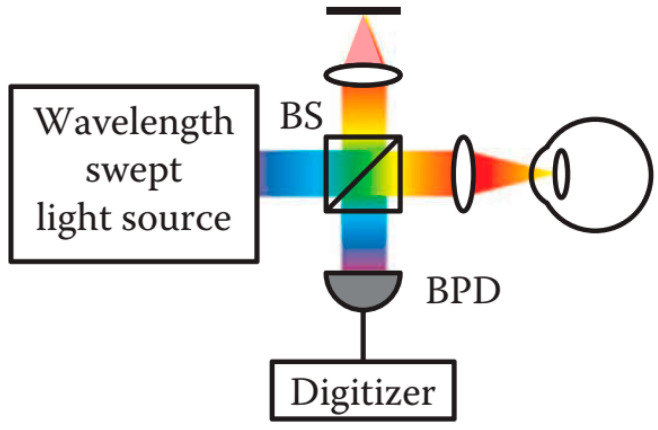
Schematic diagram of a SS-OCT system [85]. BS: Beam splitter; BPD: Balanced photodetector.

SS-OCT detects the interference signal as a function of time by using a wavelength of frequency swept light source which is different from the other schematics [113]. The setup of SS-OCT essentially consists of a Michelson interferometer with a fixed reference arm as SD-OCT. Instead of using a broadband light source and a spectrometer in SD-OCT, SS-OCT applies a narrow-band light source rapidly swept across a broad spectrum and a single or dual balanced photodetector with a high-speed A/D converter [137,162]. The swept light sources—usually as wavelength tunable lasers—sweep through multiple wavelengths over time. The consecutive spectral information is synchronously detected by a high-speed photodetector, which reconstructs the spectral fringes parameterized in time, as shown in Figure 17 [113,145,156]. The A-Scans can be generated by means of a Fourier Transform by using the conversion between time and signal intensity at a different wavelength [85]. The imaging speed in SS-OCT is determined by the sweep repetition rate of the swept light source. The spectral resolution is determined by the instantaneous coherence length of the wavelength swept light source, combined with the acquisition rate of the photodetector.

SS-OCT has many advantages over SD-OCT including reduced fringe wash-out effects, lower sensitivity attenuation, higher detection efficiency, and the ability to implement dual balanced detection [153,162]. Although SS-OCT provides improved imaging speed and higher sensitivity, its main drawback to date is the intrinsic instability of the light sources [119]. Moreover, as a point scanning OCT, SS-OCT still ultimately hits the physical boundary of the detection sensitivity when further increasing the imaging speed. The MHz swept source technology is still a challenge for clinical use. The contradiction is that the increasing speed requires an equal increase in applied optical intensity to keep the imaging sensitivity, while the optical power for in vivo imaging is strictly limited by laser safety regulations. The situation is relaxed for the parallel approaches using extended illumination, usually referred to “en-face” approaches, such as in full field OCT (FF-OCT), where higher power can be applied for the same exposure time [152].

#### 2.6.3. Full Field OCT

**Figure 18:**
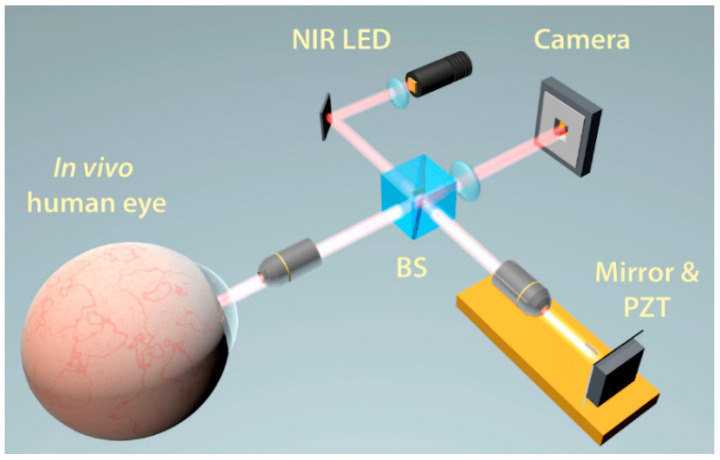
Schematic diagram of a full field OCT (FF-OCT) system [172].

FF-OCT, as a variant OCT, combines the penetration capability and high axial resolution sectioning of OCT with the high transverse resolution of confocal microscopy [163,164,165]. In contrast with the conventional OCT techniques, FF-OCT provides en-face (transverse) tomographic images directly without point-by-point raster scanning [150,163,166]. Similar to the configuration of TD-OCT using the scanning reference arm, FF-OCT typically employs a Michelson interferometer but with identical microscope objectives in both arms, commonly referred to as a Linnik interferometer [167,168]. By simply using a megapixel area CCD or CMOS camera as the detector and a white light source such as a tungsten-halogen lamp or multi-LED fibre bundle, FF-OCT enables an entire field of view imaging, approximately covering 1~2 cm^2^, through depths of hundreds of microns at the cellular level, similar to histology, but without the need for fixatives or stains. By mechanically moving the focal plane at different depths, the interferometer produces 3D tomography, as shown in Figure 18 [169,170,171].

In FF-OCT, contrary to both the confocal microscopy and conventional OCT, the isotropic resolution can achieve 1 µm or less even in strongly backscattering structures. The transverse and axial resolution are independent of each other. FF-OCT significantly improves the transverse resolution to 1 µm using medium or large numerical aperture microscope objectives, while the conventional OCT techniques employ lower aperture optics with a larger depth of field thus the lateral resolution is 5~40 µm. FF-OCT also offers the advantage of simplicity and a larger field of view compared to traditional cross-sectional OCT techniques [150,163,170]. Full field OCT was introduced in 2002 [173], and the first images of the cornea were demonstrated in 2005 [150,164,174].

Although the setup of FF-OCT is simple, it can be difficult to align the optical pathway from centimeters to less than one micrometer. In addition, the current configuration is limited for higher axial scanning speed due to its inherent sensitivity to motion. In terms of other limitations, the crucial degradation of the contrast and image resolution with the imaging depth has been hypothesized to result from multiple scattered lights. In addition, in vivo imaging is limited by the acquisition speed of the cameras currently available [144,164,175]. Table 1 provides the major pros and cons of the different categories to summarize the development of OCT. FD-OCT overcomes the limitations of low scanning speed and low detection sensitivity in TD-OCT. FD-OCT still utilizes the point scanning scheme. FF-OCT adopts area scanning scheme and realizes an en-face, broadband interferometric approach to image the anterior segment with the highest sensitivity. Although commercial equipment for anterior segment imaging in the human eye using FF-OCT is unavailable at present. The FF-OCT based approach would be applied to in vivo anterior segment imaging [150,172]. The most notable advantage of OCT is the complete decoupling of the axial resolution from transverse resolution [176]. The axial (depth) resolution of OCT is defined by the coherence length of the light source. The transverse resolution of TD-OCT and FD-OCT is determined by the focal spot size. In contrast, the transverse resolution is determined by the confocal microscopy scheme in FF-OCT [166]. However, in the Scanning-slit imaging and Scheimpflug imaging, the axial and transverse resolutions are both dependent on the focusing conditions such as the focal depth and field of view.

**Figure 19:**
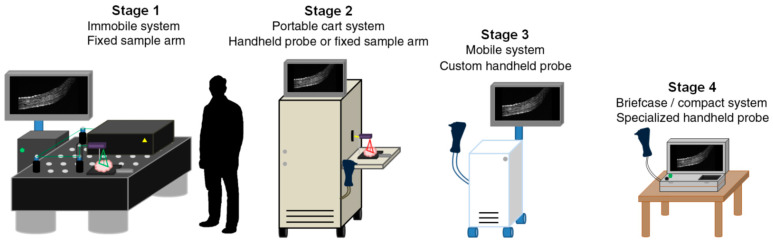
The trend of the development of OCT systems [147].

OCT can currently achieve higher imaging speed and deeper penetration depth over the other techniques, which offers the opportunity for dynamic investigation [138]. Figure 19 shows many advances in handheld OCT techniques [147]. Additionally, visible-light optical coherence tomography (vis-OCT) is an emerging imaging modality [177] which have already been applied in ultrahigh resolution retinal imaging [178].

One of the limitations of the most typical configuration of OCT systems is the presence of fan (or field) distortion and optical distortion. As a result, when imaging perfectly flat surfaces, curved results would be obtained [179]. Fan distortion is inherent to the scanning architecture of the system [20]. Fan distortion could be minimized by adjusting the position of the objective lens with respect to scanners [113], using the images taken axially around the confocal position of the beam [180] and executing correction algorithms based on ray propagation, Snell law, and Fermat principle [181], etc. Optical distortion is produced while imaging through the structures with varying refractive index [182]. Quantitative geometrical structures can only be retrieved accurately upon the correction of the distortions [119]. 2D corrections of optical distortion in OCT images could be implemented based on Fermat’s principle [180]. A method consisting of 3D ray tracing through the different surfaces, following denoising, segmentation of the surfaces, Delaunay representation of the surfaces, and application of fan distortion correction for 3D correction of optical distortion in OCT images was proposed [181]. Another limitation is that the saturation of the A-Scan signals occurs in point scanning OCT due to the overlay of the back-scattered and the back-reflected light. The back-scattered light from tissues is exclusively detected by OCT systems under ideal conditions. However, both back-scattered as well as back-reflected light are usually detected for some ocular surfaces, particularly for the ones normal to the optical axis of the systems, such as the corneal apex. Furthermore, OCT is unable to see through opaque media such as the iris [45,154].

Additionally, the impact of motion artifacts cannot be ignored in OCT. Speckle is one of the most devastating artifacts in OCT images. When the scatterers in anterior segment are of the same scale or smaller than the central wavelength of the light source, the interference of several partial waves produces a phenomenon known as speckle [183]. Methods for speckle reduction are divided into two main categories: Hardware-based methods and software-based methods [184]. The main hardware-based speckle reduction methods are compounding techniques where multiple decorrelated frames are averaged, such as spatial-compounding, angular-compounding, polarization-compounding, and frequency-compounding [183]. Software-based approaches are to develop digital filter algorithms to process the images. The goal of a speckle reduction algorithm is to deconvolve the noise from the original image. Recently, the deconvolution problem further advances by using machine learning algorithms. For instance, a cluster-based speckle reduction framework (CSRF) combining the feature extraction, k-means clustering, median filter, and despeckling was proposed [184]. Additionally, an expandable learnable despeckling framework to organize the despeckling algorithms in an intelligent manner was presented [185].

The comparison of the mentioned measuring approaches is shown in Table 2, including the measuring strategy, specifications, and major limitations. As more and more sophisticated technologies are developed, the noninvasive measurement of the anterior segment is implemented with higher speed, finer resolution, and larger range. Along with the development of the related technologies, novel optical imaging systems would be further evolved for in vivo measurement of the anterior segment or even the whole eye.

There are other approaches that are not discussed, such as the magnetic resonance imaging (MRI) [187] and photoacoustic imaging (PA imaging) [188]. In contrast to Scheimpflug imaging, MRI provides lower-resolution images of the adult eye but is undistorted [91]. The MRI images are always used to construct a computer eye model [14,17,187]. However, the data from Scheimpflug imaging have a consistently smaller error compared to data obtained with MRI [17,189,190]. Additionally, the images acquired by using MRI is approximately 3 min [91]. Photoacoustic (PA) imaging is a potential research tool and medical screening device for investigations and diagnoses of ocular diseases by measuring the optical absorption properties of the tissues [191]. Nearly all anterior segment PA images have been obtained with a mechanical-scanning optical resolution PA microscopy (OR-PAM) [191]. It can take more than 30 min for obtaining a 2 × 2 mm image by using OR-PAM [192]. In addition, a water tank is needed to achieve ultrasonic coupling between the eye and the ultrasonic detector [191]. Therefore, PA-imaging systems using OR-PAM are not suitable for clinics, which requires fast imaging speeds and improved ultrasonic detection [191]. The time consumption and lower resolution are the major drawbacks of MRI and PA imaging, which confine their application in vivo geometric measurement of the anterior segment at present. Recently, the resolution and speed improvement methods of MRI and PA imaging are still under investigation, such as using the magic-angle enhancement effect to improve MRI sensitivity [193] and developing a prototype ocular imaging system that integrates optical-resolution photoacoustic microscopy and high-frequency ultrasound imaging to achieve 6.5 min for 2 × 2 mm anterior segment imaging [194].

## 3. Challenges and Perspectives

Although the approaches mentioned above possess different advantages and also suffer different drawbacks, it should be noted that there are two main common and natural challenges for precisely measuring the geometric shapes of the anterior segment and other human eye tissues. One is the motion artefacts caused by the inevitable and random human eye movement. The other is the serious multiple scattering effects in intraocular turbid media.

### 3.1. Motion Artefacts

For the noninvasive in vivo measurement of the human eye, any eye movement during imaging would corrupt the data, making the 3D reconstruction of the anterior segment unreliable [195,196,197,198]. This problem was recognized early in 1796 when Ramsden introduced a method to eliminate the inaccuracies generated by eye movements and observer estimations as the inventor of the early-stage keratometer [23]. Sequentially scanning is now the common scheme of the technologies for anterior segment measurement. The motion artefacts during the scanning process remains one of the major challenges for precisely measuring the ocular structures, especially for investigating the intraocular tissues. There are several effective strategies to overcome the impacts of the motion artefacts.

- Minimizing the motion

The eye movement is classified into two domains: Voluntary and involuntary. Gazing is the most basic voluntary eye movement, including the eye and head motion. Head motion can be minimized by placing the subject’s head in a restraining fixture, such as a chin cup and forehead rest. Voluntary eye motion can be reduced by asking the subject to fixate on and track a stationary target [76]. However, the eyes remain in motion even during conscious fixation, referred as the involuntary motion [76,199]. The involuntary eye motion could result in around 30 arc min/s movement of the fixated point [199]. Thus, the motion artefacts caused by the involuntary eye movement is inevitable and random. The imaging resolution and measuring accuracy would be significantly degraded.

- Appending additional information or increasing the acquisition speed

To improve the accuracy of the image registration and the further 3D reconstruction, appending additional information is an effective practice. The Orbscan system relies on the Placido disk images to assist in the image registration for the 3D reconstruction [23]. The Pentacam system utilizes the rotational scanning around a single point of fixation as the patient focuses on a central light source. In this case, the artifact created by small movements during image acquisition could be reduced [23]. In addition, the Pentacam system is equipped with a second camera to track the eye movements [34,200,201]. The correction of the motion artifacts counts on the tracking information. Galilei dual Scheimpflug analyzer simply averages the data from each view to compensate for the unintentional misalignment produced by the living human eyes movement [186]. However, coupling the imaging to eye tracking results in higher production costs and increased scanning time [199]. Short image acquisition times have the natural advantage to reduce the impact of the motion artefacts incurred by movements of the globe, eyelids, and head [111]. Thus, the common solution is to increase the acquisition speed [179].

- Correcting motion artifacts in the image registration

Even though the faster scan leads to fewer distortion caused by the motion artefacts, correcting the motion artifacts after imaging in the image registration is still indispensable to achieve higher accuracy. The correction approaches have been addressed to some extent [197,198,199].

The fixation of the head and eye is the preset step. The image registration is the post-processing step. Back to the discussion on the perspectives of the measuring technologies, as motion errors are inevitable with any sequentially scanning scheme, increasing the acquisition speed is the certain approach to minimize the impact of degrading the resolution. Novel optical imaging systems should achieve further innovations in core techniques including: (a) New light sources, such as reliable broadband low coherence and high-speed tunable light sources; (b) high-speed acquisition and high-sensitivity detection systems, such as faster cameras or photodetectors and adaptive optics systems; (c) new methods for real-time signal processing, such as the introduction of GPU techniques [158].

Moreover, for in vivo 3D measurement of the anterior segment or other ocular structures, the speed, resolution, and range are strongly linked. There is always a trade-off among the imaging speed, resolution, and range. Expanding the imaging range, including both the penetration depth and field of view, is the essential requirement for whole eye detection. High speed, high resolution, and large range imaging would create opportunities for the advancement of the ophthalmology as well as other fields, such as customized eye modeling, understanding of the dynamic structural changes in the crystalline lens during accommodation, fully characterizing the performance of IOLs, investigating the corneal biomechanical properties, etc. [20,152,162].

### 3.2. Intraocular Multiple Scattering Effects

**Figure 20:**
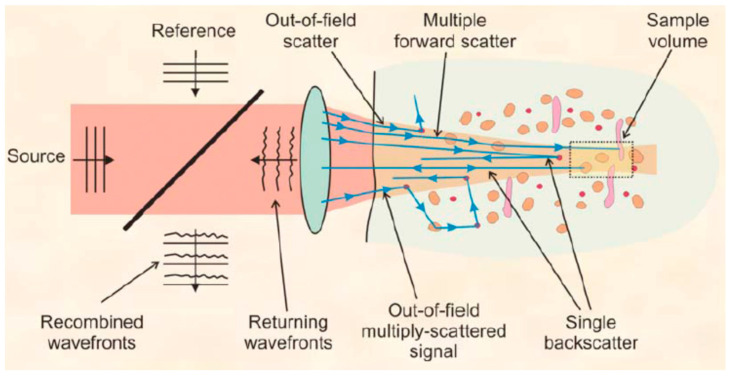
Single and multiple back scattered light [119].

Furthermore, accurate imaging through intraocular turbid media faces serious multiple scattering effects from surrounding tissues occur, as shown in Figure 20. The back-scattered light depends on the scattering properties of the tissue material, such as the reflectivity, size, position shape, and concentration of the backscatter elements. Multiple scattering occurs in the turbid media. This phenomenon would bring out serious noise during the imaging, which is another key challenge to provide a quantitative geometric measurement of the anterior segment and the other ocular tissues [144,202,203].

Advanced computational imaging technology [204,205,206] provides a possible way to overcome the limitations of conventional optics in extreme conditions and solve the multiple scattering effects in the image formation process. Tomographic imaging is the key application of Computational Imaging technology. Computational Imaging refers to the process of indirectly forming images by using a tight integration of the sensing hardware and the computation algorithms rely on a significant amount of computing. It allows to break through physical boundaries of traditional optical systems, such as numerical aperture or even obliterates the need for optical elements, resulting in the imaging systems with significantly enhanced capabilities, such as super resolution, additional information such as optical phase, and the real-time control of the light transport.

## 4. Conclusions

A great variety of technologies can be employed to perform the analysis of the anterior segment, evolving from qualitative analysis to quantitative measurement, and then from topography to tomography. The technologies applied in current commercial systems include Placido disk, FTP, Scanning-slit, Scheimpflug Imaging, UBM, and various types of OCT. Placido disk and FTP only investigate the geometry of the anterior corneal surface, while the others can be capable of 3D imaging the anterior segment by using the sequential scanning strategy. UBM and OCT are further suitable for retinal imaging, which means the whole eye segment imaging can be realized.

The development of the noninvasive, hybrid, miniaturized, and portable even handheld systems would benefit the in vivo whole eye detection. For in vivo measurement of the anterior segment, there are two main challenges that need to be addressed to achieve a high speed, fine resolution and large range imaging. One is the inevitable motion artefacts. The other is the serious intraocular multiple scattering effects. Increasing the acquisition speed is the effective approach to minimize the impact caused by the motion artefacts. It is suggested that computational imaging technology would provide a possible way to eliminate the multiple scattering effects. Further innovations in core techniques are also outlined in this paper.

## Figures and Tables

**Table 1:** The pros and cons of the different categories of OCT.

OCT Categories	Pros.	Cons.
TD-OCT	simple construction and signal processing method	low scanning speed and low detection sensitivity
SD-OCT	significant detection efficiency	strong SNR roll-off in depth and low detection resolution of the spectral distribution
SS-OCT	reduced fringe wash-out effects, lower sensitivity attenuation, higher detection efficiency, improved imaging speed and higher sensitivity	the intrinsic instability of the light sources and still adopt the point scanning scheme
FF-OCT	en-face (transverse) tomographic images directly without point-by-point raster scanning, simplicity and a larger field of view	difficult to align the optical pathway, inherent sensitivity to motion, the crucial degradation of the contrast and image resolution with the imaging depth

**Table 2:** The comparison of the mentioned measuring approaches.

Measuring Strategy	Technologies	Speed	Resolution	Covering Area	Penetration Depth	Major Limitations
Pattern projection	Placido disk *^a^*	tens of ms/image *^b^*	10 μm	14 mm in diameter	only the anterior corneal surface	Surface reconstruction of the irregular cornea fails as a result of that the rings or raster patterns could merge or cross in this case.
	Rasterstereography [49,111]	tens of ms/image *^b^*	4 μm	over 12 mm in diameter		
Interferometry	Holographic technique [53,55]	1 ms	0.1 μm	entire corneal surface		The hologram is particularly easy to be affected by the vibrations and air turbulence.
	Moiré technique [67]	1 s	2.6 μm	7 mm in square		The fine structures or those with a larger gradient on the cornea would be erased due to the inevitable application of the low-pass filter in the Moiré technique, TGI, and FTP.
	TGI [76]	tens of ms/image *^b^*	6 μm	6 mm in square		
	FTP [80]	<1 s	<10 μm	20 mm in diameter		
Parallel line scanning	Scanning-slit [186]	1.5 s	>10 μm	11 mm in diameter	anterior segment	Utilizing parallel line scanning causes difficulty in the image registration due to the lack of shared points during scanning. The depth of focus is so limited that the imaging quality of the lens is poor.
Rotational line scanning	Scheimpflug imaging [10,186]	1~2 s	<10 μm	14~16 mm in diameter	anterior segment	Visualization of the entire lens and anterior chamber is inaccessible.
Point scanning	UBM [45,126]	50~100 ms/B-Scan	20~50 μm, 150 μm *^c^*	over 20 mm in diameter	from the anterior segment to the retina *^d^*	As an immersion technique, the contact and time-consuming nature limit its application.
	TD-OCT [10,137]	2000 A-Scans/s	18 μm	16 mm in width		Quantitative measurement can only be retrieved accurately upon the correction of the fan and optical distortions. OCT is unable to see through the opaque tissues.
	SD-OCT [10,137]	>300,000 A-Scans/s	5 μm	13 mm in width		
	SS-OCT [10,137]	>2,000,000 A-Scans / s	8 μm	12 mm in width		
En-face tomographic imaging	FF-OCT [150,163,172]	tens of ms/image *^b^*	1 μm	1~2 cm^2^		

*^a^* The specifications of ATLAS 9000 system (Carl Zeiss Meditec) using the Placido disk are quoted; *^b^* The imaging process is a single shot by the CCD or CMOS cameras. The speed is dependent on the frame rate of the camera; *^c^* The resolution using 10 MHz ultrasound is 150 µm, while the one using 50~100 MHz ultrasound is 20~50 μm; *^d^* UBM is capable of imaging the retina only when the 10 MHz ultrasound is applied. The 50~100 MHz ultrasound is suitable for imaging the anterior segment. Analogous to UBM, OCT uses 850 nm light to investigate the retina and 1310 nm light to depict the anterior segment typically.
